# Body Composition Measurement Improved Performance of GLIM Criteria in Diagnosing Malnutrition Compared to PG-SGA in Ambulatory Cancer Patients: A Prospective Cross-Sectional Study

**DOI:** 10.3390/nu13082744

**Published:** 2021-08-10

**Authors:** Yanfei Wang, Xiaoyan Chen, Yunyi Wang, Ziqi Liu, Yu Fang, Zhi Peng, Wei Liu

**Affiliations:** 1 Key Laboratory of Carcinogenesis and Translational Research (Ministry of Education/Beijing), Day Oncology Unit, Peking University Cancer Hospital & Institute, Beijing 100142, China; yanfeiwang@bjmu.edu.cn (Y.W.); xiaoyan9312@126.com (X.C.); 2 Key Laboratory of Carcinogenesis and Translational Research (Ministry of Education/Beijing), Supportive Care Department, Peking University Cancer Hospital & Institute, Beijing 100142, China; Beijingwangyunyi@163.com; 3 Key Laboratory of Carcinogenesis and Translational Research (Ministry of Education/Beijing), Department of Nutrition, Peking University Cancer Hospital & Institute, Beijing 100142, China; liuziqi@bjmu.edu.cn (Z.L.); fangyu777@bjmu.edu.cn (Y.F.); zhipeng@bjmu.edu.cn (Z.P.); 4 Key Laboratory of Carcinogenesis and Translational Research (Ministry of Education/Beijing), Department of Gastrointestinal Oncology, Peking University Cancer Hospital & Institute, Beijing 100142, China

**Keywords:** malnutrition, GLIM, PG-SGA, body composition measurement, muscle mass reduction

## Abstract

Background and aims: Muscle mass reduction (MMR) is one of the three etiologic criteria in the Global Leadership Initiative on Malnutrition (GLIM) framework. This study aimed to evaluate the value of MMR in GLIM criteria among ambulatory cancer patients. Methods: A single-center prospective cross-sectional study was conducted. All participants underwent calf circumference (CC) measurement and body composition measurement by bioelectrical impedance analysis (BIA). MMR was identified by CC, fat-free mass index (FFMI), appendicular skeletal muscle index (ASMI), or combinations of the above three indicators. Patients-generated Subjective Global Assessment (PG-SGA) was used as the comparator. Results: A total of 562 cancer patients receiving intravenous treatment were evaluated. Of the participants, 62.8% (355/562) were male. The median age of the patients was 59.0 years (range, 21–82 y). The median BMI was 22.8 kg/m^2^ (range, 14.6–34.5 kg/m^2^). A total of 41.8% of patients were evaluated as malnutrition (PG-SGA ≥ 4), and 11.9% were diagnosed with severe malnutrition (PG-SGA ≥ 9). For the GLIM criteria, the prevalence of malnutrition was 26.9%, and severe malnutrition was 12.3%. For all criteria combinations of GLIM together versus PG-SGA, sensitivity was 60.4% (53.8–66.7), specificity was 97.9% (95.4–99.1), while the accordance between GLIM and PG-SGA was moderate (κ = 0.614). The performance of the GLIM worsened when MMR was excluded (κ = 0.515), with reduced sensitivity (50.2% (43.7–56.8)) and the same specificity (97.9% (95.4–99.1)). Including FFMI and ASMI by BIA can further improve the performance of GLIM than using CC alone (κ = 0.614 vs. κ = 0.565). Conclusions: It is important to include MMR in the GLIM framework. Using body composition measurement further improves the performance of the GLIM criteria than using anthropometric measurement alone.

## 1. Introduction

Cancer patients are at high risk of malnutrition. It is estimated that approximately 30–90% of cancer patients suffer from malnutrition due to either the physical and metabolic effects of cancer or the adverse effects of anticancer treatments [1,2]. Malnutrition is associated with reduced treatment effectiveness [3,4], increased treatment toxicities [5], impaired functional status and quality of life [6], higher healthcare costs [7], and poorer survival [8,9,10]. Routine nutritional screening and assessment are suggested in different clinical settings considering the adverse impacts of malnutrition on cancer patients.

Operational diagnostic criteria for the Global Leadership Initiative on Malnutrition (GLIM) were developed from 2016 to 2018 by several global clinical nutrition societies to standardize malnutrition diagnosis in different clinical settings worldwide [11,12]. Since the GLIM criteria are consensus-based, it is necessary to confirm their validity and refine the operational criteria [13,14]. 

The Patient-generated Subjective Global Assessment (PG-SGA) is a nutrition assessment tool adapted for oncology patients from the Subjective Global Assessment (SGA) tool [15]. It is commonly used among oncology patients worldwide and is recommended by the American Dietetic Association (ADA) [16] and the Chinese Society Oncological Nutrition supportive Care (CSONSC). According to recent published validation guidance on GLIM criteria [13,14], PG-SGA can be used as a secondary reference criterion to diagnose malnutrition in cancer patients. 

Muscle mass reduction (MMR) is one of the three phenotypic criteria of the GLIM framework. However, MMR is often omitted in recent literature, which aims to validate GLIM criteria [17,18]. MMR is supposed to be accessed by validated body composition measuring techniques such as fact-free mass index (FFMI) by dual-energy absorptiometry (DXA) or corresponding standards using other body composition methods like bioelectrical impedance analysis (BIA), computed tomography (CT), or magnetic resonance imaging (MRI) [11]. Since the above methods are not often available in most clinical settings for nutritional assessment worldwide, physical examination or anthropometric measures of calf or arm muscle circumference are included as alternative measures [11,12]. To our knowledge, no studies had accessed the difference between using validated body composition measuring techniques, physical examination, or anthropometric measures up to when we constructed this manuscript. 

The current study aimed to evaluate the value of MMR in the GLIM framework and compare different methods of defining MMR in an ambulatory clinical setting. 

## 2. Materials and Methods

### 2.1. Study Design

Our single-center cross-sectional, observational study was conducted at the Day Oncology Unit in Peking University Cancer Hospital, Beijing, China, spanning 3 weeks from 11 November 2020 to 10 December 2020. 

### 2.2. Participants

The eligibility criteria included: (1) diagnosed with cancer by pathology, (2) age ≥18 y, (3) receiving in-chair intravenous treatment, and (4) having normal cognitive function. Patients were excluded from the study if they were: (1) in poor performance status, namely Eastern Cooperative Oncology Group (ECOG) score > 2; (2) unable to stand up due to illness, or (3) unwilling to participate in the study.

### 2.3. Data Collection 

Before the data collection, three doctors and one nutritionist were trained by the same instructor. The training session began with the content and standard procedures of the data collection, followed by a practice session. Any confusion and discrepancies were discussed and solved during the session. The trained doctors and nutritionist completed the data collection by applying a structured questionnaire within 4 h of hospital admission. 

The questionnaire included all items from the PG-SGA and GLIM. Decreased food intake was asked for the past week, 2 weeks, and 1 month based on patients’ estimation of reduction in general food intake. Body weight in the past (0.5, 1, 2, 3, or 6 months) was recorded based on patients’ self-reporting and electronic medical records (if available), and then the percentage of unintentional weight loss was calculated. 

The collected data also included patients’ general information, plasma C-reactive protein (CRP) values, anthropometric measurements, and body composition measurement.

Patients’ general information, including age, sex, tumor site, tumor stage, comorbidities and treatment, was obtained from medical records. 

CRP was tested using fasting blood, and the values within 1 week of admission were obtained from electrical medical records (if available).

The anthropometric measurement included body weight, height, body mass index (BMI), percentage of weight loss, and calf circumference (CC). The patient’s body weight and height were measured in light indoor clothing without shoes at the time of admission. BMI was calculated as the weight (kg)/height (m)^2^. The CC of both sides were measured using a flexible and non-elastic tape, and the smaller value was recorded for use. 

All patients underwent human body composition measurement by BIA using InBody770 (InBody Co., Ltd., Cheonan, Korea) upon entering the ward before receiving intravenous treatment. Inbody770 reports FFMI and appendicular skeletal muscle index (ASMI) values.

The nutritional diagnosis was made during data analysis when PG-SGA and CLIM criteria items were extracted from collected data. 

### 2.4. PG-SGA

Nutritional status was assessed with the PG-SGA. PG-SGA consists of two sections, namely patient- and clinician-completed components. The patient-completed component includes four aspects: weight loss, nutritional impact symptoms, food intake, and functional capacity. The clinician-completed component assesses four aspects: disease situation, age, metabolic stress, and physical examination. Based on the above assessments, patients were classified as well-nourished (PG-SGA: A, score 0–1), suspected of being or moderately malnourished (PG-SGA: B, score 2–8), or severely malnourished (PG-SGA: C, score ≥9) [15]. In order to guide clinical practice, category B can be further divided into suspicious or mild malnutrition (score 2–3) and moderate malnutrition (score 4–8); the latter requires nutritional intervention and symptomatic treatment [19]. In this study, patients with PG-SGA score ≥4 were diagnosed with malnutrition, and those with PG-SGA score ≥9 (i.e., category C) were identified as having severe malnutrition. 

### 2.5. GLIM Criteria

#### 2.5.1. Step 1: Nutrition Screening

The malnutrition universal screening tool (MUST) was used to assess malnutrition risk as the first step of the GLIM framework followed by the diagnosis of malnutrition. 

The MUST considers parameters including BMI, unintentional weight loss, and acute disease comprising nutritional intake for >5 d. MUST scores are rated as described in the literature, with a score of 0 indicating low risk, 1 indicating medium risk, and 2 indicating high risk [20]. In this study, patients with MUST scores of medium or high risk were considered at risk of malnutrition and further evaluated by GLIM criteria.

#### 2.5.2. Step 2: Assessment for Diagnosis

The GLIM criteria consist of three phenotypic (weight loss, low BMI, and MMR) and two etiologic (reduced food intake/assimilation and disease burden/inflammation) criteria. 

##### Phenotypic Criteria

In this study, phenotypic criteria were defined as follows:

- Weight loss: weight loss is defined as unintentional weight loss of >5% within the past 6 mo or >10% beyond 6 mo.
- Low BMI: a low BMI for Asians is considered when BMI < 18.5 kg/m^2^ if age < 70 y or BMI < 20 kg/m^2^ if age > 70 y.
- MMR: In order to evaluate the value of MMR in GLIM criteria and determine the best definition of MMR, the phenotypic criterion MMR was defined using the following eight different definitions, respectively, as follows:
  1. “excluded MMR”: MMR was not used as a phenotypic criterion.
  2. “CC”: MMR is defined as CC < 34 cm in men or CC < 33 cm for women [21];
  3. “FFMI”: MMR is identified as FFMI < 17 kg/m^2^ in men or <15 kg/m^2^ in women, as established by the European Society for Clinical Nutrition and Metabolism (ESPEN) [22];
  4. “ASMI”: the cut-off points for diagnosing MMR were set as ASMI < 7.0 kg/m^2^ in men or <5.7 kg/m^2^ in women according to the Asian Working Group for Sarcopenia (AWGS) [21];
  5. “CC+FFMI”: meeting “CC” or “FFMI”;
  6. “CC+ASMI”: meeting “CC” or “ASMI”;
  7. “FFMI+ASMI”: meeting “FFMI” or “ASMI”;
  8. “CC+FFMI+ASMI”: meeting at least one criterion of “CC”, “FFMI”, and “ASMI”.

##### Etiologic Criteria

There are two different etiologic criteria in the GLIM criteria. In this study, each etiologic criterion was defined as follows:

- Reduced intake or assimilation: Reduced intake or assimilation is defined as intake ≤ 50% of energy requirement for >1 week, or reduction for >2 weeks, or the presence of disorders which affect assimilation, or gastrointestinal symptoms which were shown in the PG-SGA questionnaire
- Disease burden or inflammation: Inflammation is identified by plasma C-reactive protein (CRP) > 8 mg/L (if available; 44 patients were tested for CRP) or current diseases/injury with which inflammation is likely to be associated with [11,12] according to medical records. Since all participants of this study were with malignant disease, malignancy was not used as an indicator for inflammation.

##### Diagnosis of Malnutrition

To diagnosis malnutrition, at least one phenotypic criterion and at least one etiologic criterion should be met. As for MMR, different definitions were used for the diagnosis of malnutrition.

#### 2.5.3. Step 3: Severity Grading of GLIM Criteria

After the diagnosis of malnutrition, the severity of malnutrition is identified by phenotypic criteria. For unintentional weight loss, the cut-off values to grade malnutrition severity were a weight loss of >10% within the past 6 months or >20% beyond 6 months, as presented in the criteria [11,12]. For low BMI, The cut-off values to grade malnutrition severity were BMI < 17.0 kg/m^2^ if age < 70 y or BMI < 17.8 kg/m^2^ if age > 70 y, according to a recent study conducted in a Japanese population [23]. Since there is no consensus on the cut-off value in grading the severity of muscle mass reduction by anthropometric measurements referring to CC, FFMI, and ASMI acquired by BIA, muscle mass reduction was not further divided into different severity grades. 

### 2.6. Ethics Statement

The study was approved by the independent institutional ethics committee of the Peking University Cancer Hospital. All enrolled participants signed informed consent for the scientific use of their data.

### 2.7. Statistical Analysis

All analyses were performed using the Statistical Package for the Social Sciences (SPSS), version 20.0 (IBM, Armonk, NY, USA). Results were considered statistically significant when the *p*-value ≤ 0.05.

Continuous variables following normal distribution were presented as mean values and standard deviations and otherwise presented as medians and quartiles. Categorical variables were presented as counts and portions. 

Statistical evaluations of the CLIM criteria compared to PG-SGA were performed. Sensitivity (SE), specificity (SP), positive predictive value (PPV), and negative predictive value (NPV) were calculated to determine the performance of different combinations of GLIM criteria in light of PG-SGA as the gold standard. Data were expressed as a percent and 95% confidence interval (CI). Validity statistics were calculated using all possible combinations of the GLIM phenotypic and etiologic criteria; each combination of two criteria, since in some clinical situation, not all data for each criterion were accessible. Comparisons were made to the criterion of malnutrition (PG-SGA score ≥ 4) or severe malnutrition (PG-SGA score ≥ 9). The agreement among the GLIM and PG-SGA was addressed by κ. κ that is >0.80 is substantial, whereas 0.61–0.80 is moderate, the presence of lower κ values brings into question the reliability of the GLIM criteria [14,24]. The rating of validity test statistics also followed recommended cut points for sensitivity and specificity: both SE and SP > 80% is ‘good’; SE or SP > 80% and both >50% is ‘fair’; SE or SP > 50% is ‘poor’ [17,25].

## 3. Results

### 3.1. Characteristics of Participants 

**Figure 1:**
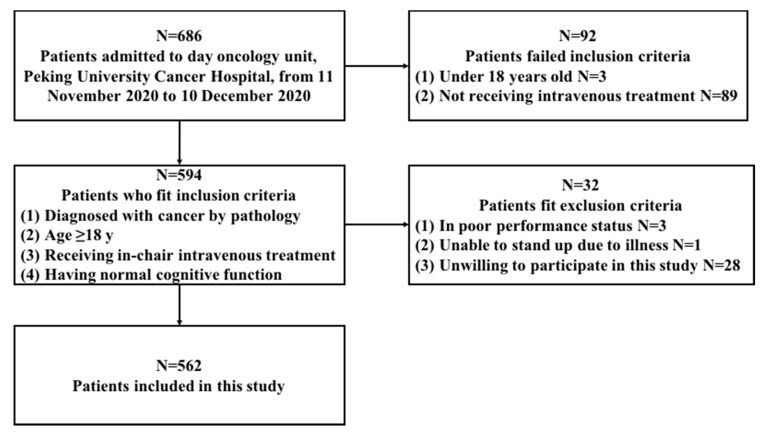
Flow chart.

A total of 686 patients were admitted to day oncology from 11 November 2020 to 10 December 2020, and 562 cancer patients participated in this study (see flow chart, Figure 1).

Of the participants, 62.8% (355/562) were male, and 37.2% (210/562) were female, with a male to female ratio of 1.69:1. The median age of the patients was 59.0 years (range, 21–82 y; the interquartile range was 52.0–65.0 y). The median BMI was 22.8 kg/m^2^ (range, 14.6–34.5 kg/m^2^; the interquartile range was 20.5–25.2 kg/m^2^). The characteristics of the patients are displayed in Table 1. 

### 3.2. Nutritional Screening and Evaluation Results of the Participants

Of the 562 patients, the prevalence of malnutrition as per scored PG-SGA ≥ 4 was 41.8% (235/562), from which severe malnutrition (scored PG-SGA ≥ 9, or PG-SGA C) was 11.9% (67/562). 

The prevalence of patients at malnutrition risk, namely MUST moderate or high risk, was 59.8% (336/562). Patients screened as a malnutrition risk were further evaluated by the GLIM criteria.

As for the GLIM criteria, the prevalence of participants meeting each criterion is shown in Table 2. The prevalence of malnutrition diagnosed by different combinations of phenotypic and etiologic criteria is shown in Table 3. 

Using all combinations of the above phenotypic and etiologic criteria, the prevalence of malnutrition diagnosed by the GLIM criteria was 26.5% (149/562), with a prevalence of severe malnutrition at 12.3% (69/562). Compare with PG-SGA, GLIM criteria underrepresented malnutrition while overrating severe malnutrition.

### 3.3. Comparision between the GLIM Criteria using different definitions of MMR and the PG-SGA

Using any phenotypic criteria combined with etiologic criteria yielded greater SE and κ compared with PG-SGA than using a single phenotypic criterion alone combined with etiologic criteria (shown in Table A1). Therefore, we compared GLIM framework in any combination of phenotypic and etiologic criteria to PG-SGA, so as to evaluate the value of MMR assessment in GLIM criteria.

Table 4 shows the SE, SP, PPV, and NPV of any combination of phenotypic and etiologic criteria of the GLIM framework in diagnosing malnutrition compared with PG-SGA. Incorporating MMR into the phenotypic criteria increased the SE of GLIM criteria while SP remained the same regardless of what definition of reduced mass was used. For different definitions of MMR, using FFMI to identify MMR had better accordance with PG-SGA in diagnosing malnutrition than using CC or ASMI (κ = 0.598, κ = 0.565, κ = 0.586, respectively, Table 4). Any combination of the indicators (CC+FFMI, CC+ASMI, FFMI+ASMI, and CC+FFMI+ASMI) further improved performance compared with using one indicator alone. When we combined CC, FFSMI, and ASMI to Aqdefine MMR, the GLIM framework had the best performance compared with PG-SGA, which had an SE of 60.4% (53.8–66.7), SP of 97.9% (95.4–99.1), and accordance with the PG-SGA was moderate (κ = 0.614). 

For diagnosing of severe malnutrition, excluding MMR or using different definitions to define MMR in GLIM criteria yielded the same results referring to SE (58.2% (45.5–69.9)), SP (93.9% (91.4–95.8)), PPV (56.5% (44.1–68.2)) and NPV (94.3% (91.8–96.1)), as well as the accordance to PG-SGA (κ = 0.515). (Table 5).

## 4. Discussion

Since the publication of the GLIM criteria in 2019, there have been emerging studies assessing the validity of the GLIM framework for diagnosing malnutrition. A big problem for validation is that there are currently no worldwide gold standards for diagnosing malnutrition. An in-depth nutrition assessment completed by experienced nutrition experts could be regarded as the semi-gold standard for validating the GLIM framework [13,14], but the specific procedures requested were still uncertain. For now, standardized tools which have been validated, such as SGA, PG-SGA, or Mini Nutritional Assessment (MNA), are still the most commonly used standard for determining the validity of GLIM [13,14,17]. Since PG-SGA is specially designed for cancer patients and has been used worldwide, we chose PG-SGA as the comparator to evaluate the performance of GLIM using different definitions of criteria.

Previous studies revealed that two-item single combinations of GLIM criteria are insufficient to diagnose malnutrition, while combining any two etiologic or phenotypic criteria greatly improves sensitivity [17], which is consistent with our result shown in Table A1. There has been strong evidence to support MMR, as one of the three phenotypic criteria, to be included in GLIM criteria. However, there is no consensus regarding measuring and defining MMR, particularly in clinical settings [11,12]. 

In published studies validating GLIM, MMR was not often included in phenotypic criteria due to its retrospective nature or condition restrictions [17,18], while in some other studies, mid-arm muscle circumstance or hand-grip strength were used to represent muscle mass reduction [9,26,27]. No studies have provided reliable body composition measurement data to validate the GLIM criteria, and it had been assumed that including FFMI into the GLIM criteria may change the SE and SP [17]. Moreover, recent literature has revealed that cancer patients with reduced FFMI identified by BIA had shorter survival (14.0 months vs. 45.1 months; *p* < 0.001) and worse quality of life than the cancer patients with normal FFMI [28], which proved the importance of assessing MMR by BIA. In this study, we applied BIA in every participant and provided precise information on MMR. 

Our study reveals that any indicator of muscle mass evaluation (CC, FFMI, ASMI) increased sensitivity of diagnosing malnutrition compared with the GLIM criteria that excluded MMR, yielding better accordance with PG-SGA; this indicated the importance of muscle mass evaluation, even using simple methods. While body composition measurement facilities such as DXA or BIA are not available in most medical centers, reliable objective and feasible measures can be used as a substitute. 

Despite the improvement in diagnosing malnutrition, including MMR assessment failed to improve the SE, SP, PPV, or NPV of GLIM criteria in diagnosing severe malnutrition; this might be explained by the fact that we did not further divide the MMR into different severity grading due to the lack of well-accepted cut-off points. Studies aiming to determine the cut-off points to grade MMR severities are warranted. 

Body composition measurement applied by BIA is more reliable than anthropometry measurements not only because it is a comprehensive evaluation of the body but also because it is an objective measure and evades interobserver error. Unsurprisingly, our study showed that either FFMI or ASMI yielded better results than CC, while the FFMI+ASMI combination further improved the sensitivity of GLIM. However, the best performance of GLIM criteria is acquired by combining body composition measurement (FFMI+ASMI) and anthropometry measurement (CC) together (κ = 0.614). Future study needs to be done in a wider population to determine the best method to define MMR. 

Our study has some strengths. As a prospective cross-sectional observational study, we recruited 562 patients in a short period of time (3 weeks) and all data for PG-SGA and GLIM were collected concurrently, which ensured that the conclusion drawn from the study was relatively more reliable. Moreover, we applied BIA in every participant and provided a precise evaluation of MMR. To the best of our knowledge, this is the first study including muscle mass evaluation by BIA to validate the GLIM criteria.

Nonetheless, there are several limitations to our study. Since it was a single-center observational study, results should be interpreted with caution. Food intake was assessed via self-report on the amount of food instead of an in-depth diet history to promote feasibility. Inflammation was assessed by increased CRP and current inflammation-associated disease or injury. However, the detection rate of CRP was low in our study since no patients presented diseases or injuries likely to be associated with inflammation except for malignant disease, and CRP was not a regular detected indicator for ambulatory cancer patients in our center; therefore, the evaluation of inflammation status is inadequate.Finally, we must contact the participant again to collect survival data revealing the value of the GLIM criteria in predicting clinical outcomes and survival. 

## 5. Conclusions

In conclusion, the prevalence of malnutrition is high among ambulatory cancer patients. Using PG-SGA as the standard, GLIM criteria had fair criterion validity for the diagnosis of malnutrition, regardless of severity status. It is important to include MMR in the GLIM framework, while body composition measurement applied by BIA further improves the performance of GLIM criteria. When validated facilities were not available, simple anthropometric measurements can be used. 

## Figures and Tables

**Table 1:** Characteristics of 562 patients with cancer enrolled in this study.

Parameter	Total *n* = 562
Age, y (median, interquartile range)	59 (52–65)
Sex, n (%)	
Male	354 (70.3)
Female	208 (37.0)
Tumor site, n (%)	
Upper Gastrointestinal tract	134 (23.8)
Colorectal	291 (51.8)
Head and neck	1 (0.2)
Lung and mediastinum	43 (7.7)
Breast	21 (3.7)
Urogenital	11 (2.0)
Gynecology	1 (0.2)
Liver, pancreas	40 (7.1)
Lymphoma	4 (0.7)
Other	16 (2.8)
Tumor stage, n (%)	
I	10 (1.8)
II	45 (8.0)
III	176 (31.3)
IV	331 (58.9)

**Table 2:** The prevalence of patients meeting each criterion of the GLIM framework.

Criteria		*n* (%)
Phenotypic Criteria		Total *n* = 562
Weight loss		227 (40.4)
Low BMI		60 (10.7)
MMR	CC	129 (23.0)
	FFMI	158 (28.1)
	ASMI	119 (21.2)
	CC+FFMI	194 (34.5)
	CC+ASMI	162 (28.8)
	FFMI+ASMI	168 (29.9)
	CC+FFMI+ASMI	197 (35.1)
Etiologic criteria		Total *n* = 562
Reduced intake or assimilation		186 (33.1)
Disease burden or inflammation	Inflammation-associated disease or injury	0 (0)
		Total *n* = 44
	Elevated plasm CRP	7 (1.2)

CC: calf circumference; FFMI: fat-free mass index; ASMI: appendicular skeletal muscle index; CRP: C-reactive protein.

**Table 3:** The prevalence of malnutrition diagnosed by different combinations of phenotypic and etiologic criteria of the GLIM framework.

Combination	Prevalence *n* (%)
Weight loss Reduced intake or assimilation	117 (20.8)
Low BMI Reduced intake or assimilation	25 (4.4)
MMR * Reduced intake or assimilation	87 (15.5)
Any phenotypic criterion Reduced intake or assimilation	148 (26.3)
Weight loss Disease burden/inflammation	1 (2.3)
Low BMI Disease burden/inflammation	0 (0)
MMR * Disease burden/inflammation	0 (0)
Any phenotypic criterion Disease burden/inflammation	44 (2.3)
Weight loss Any etiologic criterion	118 (21.0)
Low BMI Any etiologic criterion	25 (4.4)
MMR * Any etiologic criterion	87 (15.5)
Any phenotypic criterion Any etiologic criterion	149 (26.5)

BMI: body mass index; MMR: muscle mass reduction. MMR * in this table was defined by the combination of calf circumference, fat-free mass index, or appendicular skeletal muscle index.

**Table 4:** SE, SP, PPV, and NPV of GLIM using any combination of phenotypic criteria and etiologic criteria in diagnosing malnutrition compared with PG-SGA.

Definition of MMR	SE (%,95%CI)	SP (%,95%CI)	PPV (%,95%CI)	NPV (%,95%CI)	κ
Excluded MMR	50.2 (43.7–56.8)	97.9 (95.4–99.1)	94.4 (88.4–97.5)	73.2 (68.8–77.3)	0.515
CC	55.3 (48.7–61.7)	97.9 (95.4–99.1)	94.9 (89.4–97.8)	75.3 (70.9–79.3)	0.565
FFMI	58.7 (52.1–65.0)	97.9 (95.4–99.1)	95.2 (90.0–97.9)	76.7 (72.3–80.7)	0.598
ASMI	57.4 (50.8–63.8)	97.9 (95.4–99.1)	95.1 (89.7–97.8)	76.2 (71.8–80.1)	0.586
CC+FFMI	60.0 (53.4–66.3)	97.9 (95.4–99.1)	95. 3 (90.1–97.9)	77.3 (72.9–81.2)	0.610
CC+ASMI	59.1 (52.6–65.4)	97.9 (95.4–99.1)	95.2 (90.0–97.9)	76.9 (72.5–80.8)	0.602
FFMI+ASMI	59.1 (52.6–65.4)	97.9 (95.4–99.1)	95.2 (90.0–97.9)	76.9 (72.5–80.8)	0.602
CC+FFMI+ASMI	60.4 (53.8–66.7)	97.9 (95.4–99.1)	95.3 (90.2–98.0)	77.5 (73.1–81.4)	0.614

MMR: muscle mass reduction; SE: sensitivity; SP: specificity; PPV: positive predictive value; NPV: negative predictive value; CI: confidence interval; PG-SGA: Patient-generated Subjective Global Assessment; CC: calf circumference; FFMI: fat-free mass index; ASMI: appendicular skeletal muscle index.

**Table 5:** SE, SP, PPV and NPV of GLIM using any combination of phenotypic and etiologic criteria in diagnosing severe malnutrition compared with PG-SGA.

Definition of MMR	SE (%,95%CI)	SP (%,95%CI)	PPV (%,95%CI)	NPV (%,95%CI)	κ
Excluded MMR	58.2 (45.5–69.9)	93.9 (91.4–95.8)	56.5 (44.1–68.2)	94.3 (91.8–96.1)	0.515
CC	58.2 (45.5–69.9)	93.9 (91.4–95.8)	56.5 (44.1–68.2)	94.3 (91.8–96.1)	0.515
FFMI	58.2 (45.5–69.9)	93.9 (91.4–95.8)	56.5 (44.1–68.2)	94.3 (91.8–96.1)	0.515
ASMI	58.2 (45.5–69.9)	93.9 (91.4–95.8)	56.5 (44.1–68.2)	94.3 (91.8–96.1)	0.515
CC+FFMI	58.2 (45.5–69.9)	93.9 (91.4–95.8)	56.5 (44.1–68.2)	94.3 (91.8–96.1)	0.515
CC+ASMI	58.2 (45.5–69.9)	93.9 (91.4–95.8)	56.5 (44.1–68.2)	94.3 (91.8–96.1)	0.515
FFMI+ASMI	58.2 (45.5–69.9)	93.9 (91.4–95.8)	56.5 (44.1–68.2)	94.3 (91.8–96.1)	0.515
CC+FFMI+ASMI	58.2 (45.5–69.9)	93.9 (91.4–95.8)	56.5 (44.1–68.2)	94.3 (91.8–96.1)	0.515

MMR: muscle mass reduction; SE: sensitivity; SP: specificity; PPV: positive predictive value; NPV: negative predictive value; CI: confidence interval; PG-SGA: Patient-generated Subjective Global Assessment; CC: calf circumference; FFMI: fat-free mass index; ASMI: appendicular skeletal muscle index.

## Appendix A

**Table A1:** SE, SP, PPV, and NPV of GLIM using different combinations of phenotypic criteria and etiologic criteria in diagnosing malnutrition compared with PG-SGA.

Combination	SE (%,95%CI)	SP (%,95%CI)	PPV (%,95%CI)	NPV (%,95%CI)	κ
Weight loss Reduced intake or assimilation	48.5 (42.1–55.0)	98.2 (95.9–99.2)	95.1 (89.2–98.0)	72.1 (67.7–76.2)	0.488
Low BMI Reduced intake or assimilation	10.2 (6.8,15.0)	99.7 (98.0–100.0)	96.0 (77.7–99.8)	60.7 (56.4–64.8)	0.113
MMR * Reduced intake or assimilation	36.2 (30.1–42.7)	99.4 (97.6–99.9)	97.7 (91.2–99.6)	68.4 (64.0–72.5)	0.390
Any phenotypic criterion Reduced intake or assimilation	60.4 (53.8–66.7)	98.2 (95.8–99.2)	95.9 (91.0–98.3)	77.5 (73.1–81.4)	0.618
Weight loss Disease burden/inflammation	0 (0–2.0)	99.7 (98.0–100.0)	0 (0–94.5)	58.1 (53.9–62.2)	−0.004
Low BMI Disease burden/inflammation	0 (0–2.0)	100.0 (98.6–100.0)	-	-	0.000
MMR * Disease burden/inflammation	0 (0–2.0)	100.0 (98.6–100.0)	-	-	0.000
Any phenotypic criterion Disease burden/inflammation	0 (0–2.0)	99.7 (98.0–100.0)	0 (0–94.5)	58.1 (53.9–62.2)	−0.004
Weight loss Any etiologic criterion	47.2 (40.7–53.8)	97.9 (95.4–99.1)	94.1 (87.8–97.4)	72.1 (67.6–76.1)	0.485
Low BMI Any etiologic criterion	10.2 (6.8–15.0)	99.7 (98.0–100.0)	96.0 (77.7–99.8)	60.7 (56.4–64.8)	0.113
MMR * Any etiologic criterion	36.2 (30.1–42.7)	99.4 (97.6–99.9)	97.7 (91.2–99.6)	68.4 (64.0–72.5)	0.39
Any phenotypic criterion Any etiologic criterion	60.4 (53.8–66.7)	97.9 (95.4–99.1)	95.3 (90.2–97.9)	77.5 (73.1–81.4)	0.614

SE: sensitivity; SP: specificity; PPV: positive predictive value; NPV: negative predictive value; CI: confidence interval; PG-SGA: Patient-generated Subjective Global Assessment; BMI: body mass index; MMR: muscle mass reduction. * MMR in this table was defined by the combination of CC, FFMI, or ASMI.
